# Metabolic disturbances in acute pancreatitis: mechanisms and therapeutic implications

**DOI:** 10.3389/fendo.2025.1579457

**Published:** 2025-08-27

**Authors:** Chao Fang, Yingwei Ding, Xiaojun Wang, Xusheng Teng

**Affiliations:** Department of Emergency, Jinhua Municipal Central Hospital, Jinhua, China

**Keywords:** metabolic disturbances, acute pancreatitis, lipid metabolism, glucose metabolism, oxidative stress

## Abstract

Acute pancreatitis (AP) is a common inflammatory condition of the pancreas that is often associated with metabolic disturbances resulting from pancreatic injury. This review examines the intricate relationship between metabolic abnormalities, such as changes in lipid and glucose metabolism, and the pathophysiology of AP. While these metabolic disturbances do not directly cause AP, they can significantly worsen the progression and severity of the disease. For instance, hypertriglyceridemia can increase pancreatic necrosis through mechanisms like lipotoxicity and oxidative stress. Similarly, disorders in glucose metabolism can further damage pancreatic cells by heightening inflammatory responses and oxidative stress. Additionally, we investigate novel metabolic interventions, including lipase inhibitors, insulin therapy, and antioxidants, designed to address these metabolic disturbances and reduce the severity of the disease. Understanding how metabolic disturbances contribute to the progression of AP is crucial for developing effective therapeutic strategies and improving patient outcomes.

## 1 Introduction

Acute pancreatitis (AP) is a common acute abdominal disorder characterized by inflammation within the pancreas. Various factors, including gallstones, excessive alcohol consumption, certain medications, and infections, can trigger this condition. Epidemiological data show a gradual increase in the incidence of AP over recent years (1). Globally, the annual incidence of AP is estimated to range from 34 to 80 cases per 100,000 individuals (2). The epidemiological landscape of AP is changing significantly, largely due to socioeconomic development and the increasing prevalence of Westernized dietary patterns. The widespread adoption of high-fat diets and rising obesity rates are key drivers of the growing incidence of AP (3). Notably, in regions characterized by a high prevalence of obesity and diabetes, the incidence of AP is even higher, emphasizing the complex relationship between lifestyle factors and disease occurrence (4). The pathogenesis of AP involves the activation of proteolytic enzymes within pancreatic cells, leading to autodigestion of pancreatic tissue and localized injury (5). As inflammation progresses, immune cells are recruited and activated, and inflammatory cytokines are released, which further increase pancreatic injury and contribute to the development of necrosis. In recent years, the role of metabolic disturbances in AP has gained attention. As both an endocrine and exocrine organ, the pancreas plays a crucial role in metabolic processes that affect its function and modulate the inflammatory response during AP.

Metabolic dysregulation, such as disorders in lipid metabolism, glucose metabolism abnormalities, and issues with amino acid metabolism, exacerbates pancreatic injury and inflammatory responses. For instance, the presence of hyperglycemia and insulin resistance (IR) negatively impacts pancreatic cell function and worsens the clinical outcomes of AP by modulating immune responses, promoting the secretion of pro-inflammatory cytokines, and inducing apoptosis (6). Moreover, reactive oxygen species (ROS) generated by oxidative stress play a crucial role in mediating the inflammatory cascade (7), while an abnormal accumulation of fatty acids is linked to cellular injury and tissue necrosis (8).

This review aims to explore the mechanisms behind metabolic alterations in AP, focusing on how metabolic dysregulation contributes to the disease’s pathogenesis and evaluating existing metabolic intervention strategies. Metabolic disturbances significantly impair pancreatic function and alter the cellular microenvironment, leading to enhanced inflammatory responses and disease progression in AP. Understanding how metabolic dysregulation impacts AP is crucial, as it provides insight into the disease’s pathogenesis and opens up potential new avenues for clinical management.

## 2 The underlying pathological mechanisms of AP

### 2.1 The early-phase response in AP

**Figure 1:**
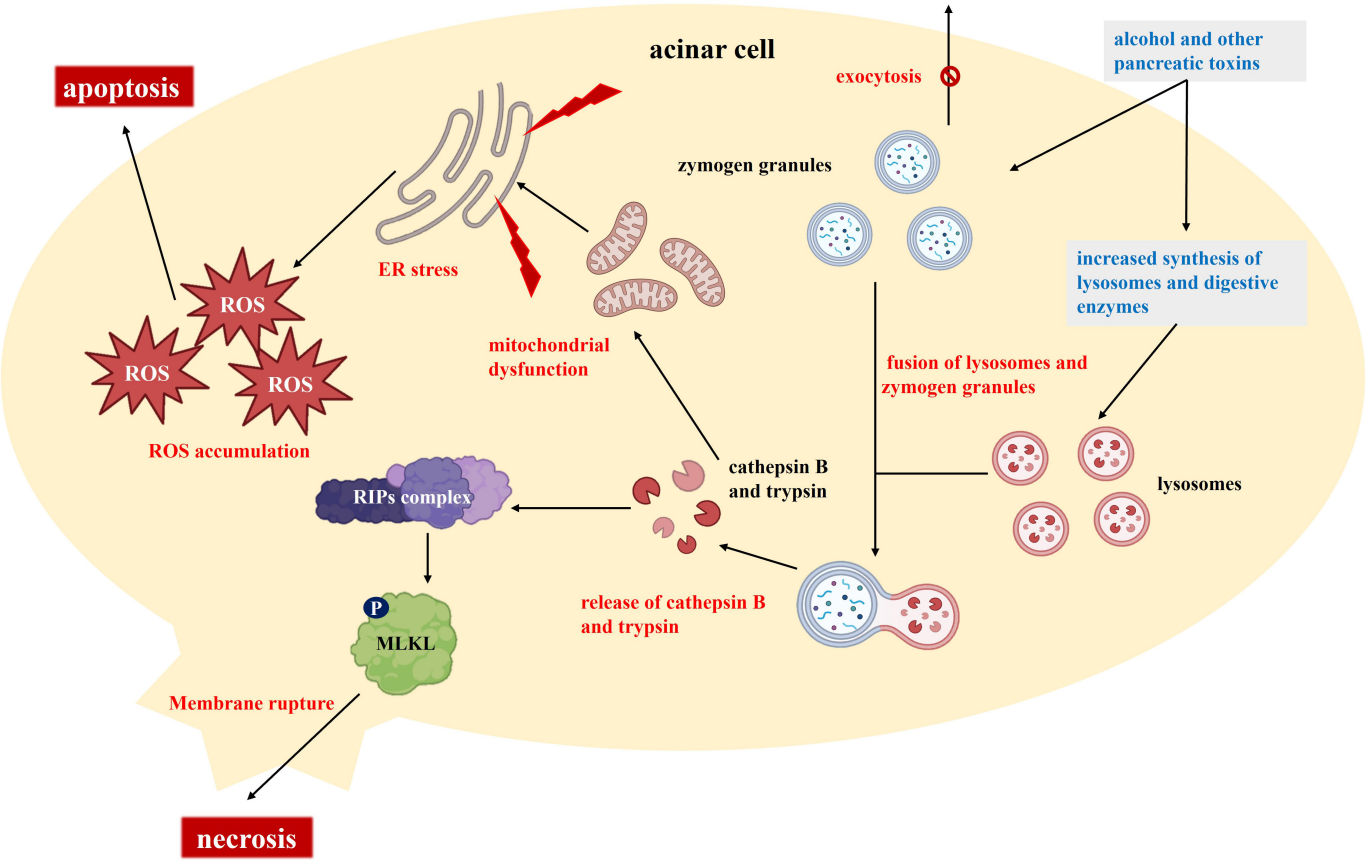
Molecular and cellular mechanisms underlying the pathogenesis of acute pancreatitis.

The initial response in AP typically begins with acinar cell injury (**Figure 1**). In acinar cells, substances like alcohol induce microtubule dysfunction, promote the synthesis of lysosomes and digestive enzymes, and disrupt the exocytosis of trypsinogen granules (9). This process results in the accumulation of enzyme granules within the cells and the fusion of lysosomes with these granules. Cathepsin B, located in lysosomes, co-localizes with trypsinogen, and both cathepsin B and activated trypsin are released into the cytoplasm (10). The release of these proteases has dual effects: it activates receptor-interacting protein kinases (RIPs) and mixed lineage kinase domain-like (MLKL), leading to cell membrane rupture and necrosis, while also causing mitochondrial dysfunction, activating endoplasmic reticulum (ER) stress, and promoting ROS accumulation (11, 12). Following acinar cell injury, extensive release of proteolytic enzymes occurs, resulting in local tissue autodigestion and necrosis while also initiating inflammatory responses. The activation of additional digestive enzymes and subsequent local cell damage further promote the release of inflammatory mediators, such as cytokines and chemokines, which increase macrophage infiltration and intensify the inflammatory response (13). In this process, oxidative stress and ER stress also contribute to cell death and the expansion of the inflammatory response.

### 2.2 Clinical presentations of AP

The clinical manifestations of AP can be classified into two categories based on the disease severity: mild and severe. Mild acute pancreatitis (MAP): Patients with MAP typically present with relatively mild symptoms, including persistent upper abdominal pain and associated gastrointestinal symptoms such as nausea and vomiting. The majority of patients with MAP can achieve rapid clinical improvement following early supportive care, which commonly includes fluid resuscitation, analgesia, and fasting (14). Severe acute pancreatitis (SAP): In contrast, SAP is frequently complicated by life-threatening conditions such as pancreatic necrosis, multi-organ dysfunction syndrome, and infection. Clinically, it is characterized by severe upper abdominal pain, fever, tachypnea, tachycardia, and even shock (15). The clinical course of SAP is often rapidly progressive, with a significantly higher mortality rate, particularly when complications arise. Owing to the heterogeneous and nonspecific nature of the early symptoms of AP, prompt diagnosis and intervention are essential for optimizing outcomes.

## 3 Metabolic disturbances in AP

AP is a complex disease characterized by a multifaceted interplay of pancreatic cell injury, enzyme activation, localized inflammatory responses, and systemic metabolic disturbances. The initial inflammatory cascade, triggered by pancreatic injury, is sustained through the activation of digestive enzymes and the subsequent release of pro-inflammatory cytokines. This process can potentially lead to pancreatic necrosis and multi-organ dysfunction syndrome. Furthermore, metabolic disturbances, particularly the dysregulation of glucose and lipid metabolism, play critical roles in the pathogenesis and progression of AP. In this section, we explored the intricate relationships between metabolic disturbances, pancreatic injury, and inflammatory responses, as well as their implications for early diagnosis and therapeutic interventions in AP.

### 3.1 Dysregulation of lipid metabolism

One pivotal factor in the pathogenesis of AP is the abnormal activation of lipase. Recent studies have highlighted the correlation between hypertriglyceridemia and necrotizing pancreatitis, a severe form of AP that is associated with high morbidity and mortality (16). Under normal physiological conditions, lipase is a digestive enzyme secreted by the pancreas that is primarily responsible for the digestion of dietary fats. However, in cases of AP, obstruction of the pancreatic duct or cellular injury can lead to the premature activation and intracellular release of lipase and other digestive enzymes, which initiates a cascade of pathological events, particularly in the presence of hypertriglyceridemia (17).

Hypertriglyceridemia can exacerbate the inflammatory response and tissue damage in AP, often leading to the development of necrotizing pancreatitis. When triglyceride (TG) levels are excessively high, pancreatic lipase breaks down TGs into free fatty acids (FFA), which can induce lipotoxicity (18). This lipotoxicity disrupts cell membrane integrity, increases intracellular calcium (Ca²^+^) levels, and NOD-like receptor thermal protein domain associated protein 3 (NLRP3) inflammasome. These events promote the production and release of pro-inflammatory cytokines (19). The accumulation of FFA also leads to oxidative stress, further damaging pancreatic cells and exacerbating tissue necrosis. The severity of hypertriglyceridemia has a positive correlation with the risk of developing necrotizing pancreatitis (20), with patients showing TG levels exceeding 1000 mg/dL being at significantly higher risk for this progression.

The aberrant activation of lipase exacerbates pancreatic injury through several key mechanisms: 1. Direct cellular damage: Lipase directly targets pancreatic cells, disrupting cell membranes and increasing cellular permeability. This process facilitates the formation of a necrosome through the binding of RIP1 and RIP3, ultimately leading to necrotic cell death (21); 2. Metabolic byproducts and inflammation: The byproducts of lipase activation, such as oleic acid (22), can trigger the release of intracellular Ca²^+^, inhibit mitochondrial complex I and V functions, and upregulate inflammatory cytokines. These changes lead to acinar cell necrosis and further intensify the inflammatory response within the pancreas. 3. Lipase can also affect local pancreatic blood flow and induce edema, contributing to further tissue necrosis and exacerbating the pathology (23).

Furthermore, it is crucial to explore the relationship between lipase activation and inflammatory pathways beyond necrosome formation, specifically the interaction with the NLRP3 inflammasome (24). The products of lipase activation, such as FFAs, can directly activate the NLRP3 inflammasome. This activation occurs when FFAs are absorbed by immune cells, leading to mitochondrial dysfunction and the production of ROS (25). Elevated levels of ROS then activate the NLRP3 inflammasome, promoting the processing and release of pro-inflammatory cytokines like IL-1β and IL-18. This creates a positive feedback loop, where the released cytokines further enhance lipase expression and activity, worsening both lipolysis and inflammation (26).

Additionally, the buildup of fatty acids is a significant pathological phenomenon in AP. During the progression of the disease, fatty acids are released in large quantities due to abnormal lipase activation. The intracellular accumulation of fatty acids can lead to multiple detrimental effects. First, FFAs create a high-oxidative state that triggers the release of calcium ions (Ca²^+^) from the ER, which increases intracellular Ca²^+^ concentrations and retroactively activates various digestive enzymes, including trypsin (27). This activation exacerbates the autodigestion of the pancreas, resulting in inflammatory responses and cell death. Fatty acid accumulation also induces ER stress, activates protein kinase Cα, and promotes the activation of activator protein-1 along with the secretion of tumor necrosis factor-α through stress-activated protein kinase signaling pathways. These changes ultimately worsen pancreatic cell injury (28). Moreover, fatty acids can impair mitochondrial function, as high concentrations of FFAs cause mitochondrial membrane depolarization and activate the mitochondrial permeability transition pore related to mitochondrial calcium overload. These alterations exacerbate pancreatic metabolic dysfunction and may lead to pancreatic failure (29).

Clinical studies have indicated that patients with hypertriglyceridemia-induced AP often experience more severe abdominal pain, higher fever, and markedly increased levels of inflammatory markers such as C-reactive protein (CRP) and interleukin-6 (IL-6) (30). These patients typically require longer hospital stays and may need intensive care. Early intervention to lower triglyceride (TG) levels, including insulin therapy, heparin infusion, or plasma exchange, has been shown to reduce the severity of the disease and decrease the risk of complications (31). For instance, insulin therapy lowers TG levels by stimulating lipoprotein lipase (LPL) activity, while heparin accelerates the breakdown of TG-rich lipoproteins (32). These interventions help alleviate the metabolic burden on the pancreas and prevent the progression of necrotizing pancreatitis.

Another important aspect of the link between hypertriglyceridemia and necrotizing pancreatitis is the role of genetic factors. Certain genetic polymorphisms, such as those in the LPL gene, can predispose individuals to hypertriglyceridemia and increase their susceptibility to SAP (33). Identifying these genetic risk factors may aid in the early detection of high-risk patients and guide personalized treatment strategies.

In summary, dysregulation of lipid metabolism, driven by the abnormal activation of lipase and the accumulation of fatty acids, plays a significant role in the pathogenesis of AP. Lipase activation leads to the release of fatty acids, which exacerbate pancreatic cell injury and accelerate inflammatory responses and cell death through multiple pathways (34). The correlation between hypertriglyceridemia and necrotizing pancreatitis emphasizes the importance of addressing metabolic disturbances in the clinical management of AP (35). Furthermore, lipid metabolic disturbances not only affect enzyme activity within the pancreas but also worsen systemic metabolic disorders, thus driving the progression of AP. Future research should further elucidate the complex interplay between lipid metabolism and AP, focusing on the molecular mechanisms underlying the relationship between hypertriglyceridemia and necrotizing pancreatitis and exploring novel therapeutic strategies to mitigate the damage caused by lipid metabolic disturbances, ultimately improving clinical outcomes in AP.

### 3.2 Dysregulation of glucose metabolism

Dysregulation of glucose metabolism is another critical component of the metabolic disturbances observed in AP. The development of AP not only impairs the exocrine digestive function of the pancreas but also disrupts its endocrine function, leading to dysregulated insulin secretion (36). Pancreatic injury results in either inhibited or aberrant insulin secretion, while inflammatory responses and metabolic disturbances reduce pancreatic sensitivity to insulin, thereby inducing IR. Research indicates that in the early stages of AP, the function of pancreatic β-cells may be suppressed by acute stress responses, resulting in reduced insulin secretion. Gao et al. (37) demonstrated that in the initial phase of AP, M1 macrophages deliver inflammatory mitochondria to pancreatic β-cells via extracellular vesicles, which subsequently fuse with normal mitochondria. This fusion process leads to iron accumulation in normal mitochondria, increased levels of lipid peroxides, and enhanced mitochondrial membrane permeability. Concurrently, damaged mitochondrial DNA fragments are released into the cytoplasm, activating the stimulator of interferon genes signaling pathway, which further induces apoptosis of pancreatic β-cells.

Systemic inflammatory responses in AP interfere with insulin action through multiple mechanisms, leading to exacerbation of IR For instance, elevated levels of IL-6 in the plasma of patients with AP are positively correlated with IR (38). IL-6 not only inhibits non-oxidative glucose metabolism but also suppresses LPL activity, leading to a continuous increase in plasma TG levels (39). Moreover, IL-6 activates suppressor of cytokine signaling proteins, which block the activation of insulin receptor transcription, reducing the responsiveness of target tissues such as muscle, fat, and liver to insulin. This results in disordered glucose metabolism and exacerbates hyperglycemia (40).

Moreover, adipokines play a significant role in linking obesity to pancreatitis severity. Adipokines such as leptin and adiponectin are involved in IR (41). Leptin, which is elevated in obesity, has pro-inflammatory effects and can exacerbate pancreatic injury. It can activate the NLRP3 inflammasome and promote the release of inflammatory cytokines, thereby contributing to IR and pancreatic inflammation (42). On the other hand, adiponectin has anti-inflammatory and insulin-sensitizing effects. Its levels are typically reduced in obesity, leading to decreased protection against inflammation and IR (43). The imbalance between leptin and adiponectin in obesity can worsen the severity of AP.

Disordered glucose metabolism is closely linked to the severity of AP. During the course of the disease, glucose metabolic disturbances are typically accompanied by pancreatic dysfunction. The presence of hyperglycemia and IR, particularly under the influence of inflammatory responses, exacerbates pancreatic cell injury. Studies have shown that in patients with AP, the degree of glucose metabolic disturbances is positively correlated with the intensity of the inflammatory response (44). Elevated blood glucose levels and IR are often important indicators of disease exacerbation in AP. In severe cases, damage to pancreatic β-cells and worsening IR lead to more glucose metabolic disturbances, resulting in persistent hyperglycemia. This hyperglycemia not only further exacerbates pancreatic inflammation but also affects the function of other organs, increasing the risk of complications in AP (45).

In conclusion, hyperglycemia, IR, abnormal insulin secretion from pancreatic β-cells, and glucose metabolic disturbances are closely interrelated and interact synergistically to exacerbate disease severity in AP. Controlling blood glucose levels, improving insulin sensitivity, and restoring pancreatic islet function are essential therapeutic strategies for managing AP. Future research should further elucidate the relationship between glucose metabolism and pancreatic injury, including the role of adipokines in obesity-related pancreatitis, and develop targeted therapeutic strategies to alleviate metabolic disturbances in patients with AP, thereby improving clinical outcomes.

### 3.3 Amino acid metabolism and protein synthesis

In AP, the inhibition of protein synthesis and the promotion of protein degradation are crucial processes. When AP occurs, pancreatic cells experience severe metabolic stress, especially after inflammatory responses are activated (46). At this stage, pancreatic protein synthesis is often inhibited, primarily due to endoplasmic ER stress, which suppresses protein synthesis in acinar cells through three main signaling pathways.

#### 3.1.1 The protein kinase R-like endoplasmic reticulum kinase signaling pathway

PERK, a serine/threonine protein kinase, catalyzes the phosphorylation of the eukaryotic initiation factor 2α (eIF2α) family of proteins. This action blocks downstream mRNA translation on a large scale, reducing the protein load on the ER (47). In studies involving rats with AP, ER stress can promote caspase-1-dependent pyroptosis in acinar cells via the PERK pathway, intensifying the inflammatory response (48).

#### 3.1.2 The inositol-requiring enzyme 1α signaling pathway

When large quantities of misfolded proteins accumulate in the ER, IRE1α is oligomerized and activated (49). As a bifunctional transmembrane kinase/RNase, IRE1α can cause extensive mRNA degradation to alleviate or terminate ER stress and restore cellular homeostasis (50). During AP, the release of large amounts of FFA activates the IRE1α enzyme, which mediates the splicing of X-box binding protein 1 (XBP1) mRNA. The resulting transcriptional activator, Xbp1s, helps relieve ER stress and protect acinar cells from necrosis (51).

#### 3.1.3 The activating transcription factor 6 signaling pathway

ATF6, a single-pass type II transmembrane protein, has a cytoplasmic domain composed of 370–380 amino acids, followed by a 21-amino-acid transmembrane domain and a cytoplasmic domain of 270 amino acids projecting into the ER lumen (52). When unfolded proteins accumulate in the ER, ATF6 is released and enters the nucleus, activating the transcription of multiple chaperone molecules. This process destabilizes and promotes the degradation of misfolded proteins (53). Tan et al. (54) demonstrated that increased expression of ATF6 was associated with elevated apoptosis, ER, and mitochondrial disorder in pancreatic tissues from SAP patients and humanized serine protease 1 (PRSS1) transgenic mice. Knockout of ATF6 in SAP mice has been shown to attenuate acinar injury, apoptosis, and ER disorder.

ER stress is essentially a protective mechanism that reduces new protein production to alleviate the ER burden and prevent the accumulation of unfolded proteins (55). However, this inhibitory response may also negatively impact pancreatic cell repair and functional recovery, particularly in the later stages of AP (56). Prolonged inhibition of protein synthesis can diminish the repair capacity of pancreatic cells, obstructing the effective recovery of damaged pancreatic tissue and worsening the condition (57).

Protein catabolism is also crucial in AP. Due to pancreatic cell damage and inhibited protein synthesis, the pancreas activates the ubiquitin-proteasome pathway (UPS) and autophagy to process damaged or accumulated proteins (58). For instance, vacuole membrane protein 1 (VMP1), a multi-transmembrane protein in the ER, regulates autophagy by promoting autophagosome closure (58). Studies have shown that in trypsinogen-induced pancreatitis models, VMP1 interacts with the selective autophagy receptor sequestosome 1 (SQSTM1), which removes damaged zymogen granules and prevents premature intracellular trypsinogen activation (59). Protein degradation maintains intracellular homeostasis by breaking down unnecessary or damaged components. In AP, this process eliminates abnormally folded proteins, preventing further cellular damage (60). However, excessive protein degradation, especially the over-release of cytokines and proteases, can intensify inflammation and cell death, leading to further tissue destruction. Thus, the balance between protein synthesis inhibition and degradation is critical in determining the severity of pancreatic inflammation and the pancreas’ repair capacity (61).

Abnormal amino acid metabolism is another key factor affecting pancreatic repair and injury in AP. Amino acids, the building blocks of proteins, are also key metabolic regulators (62). During AP, amino acid metabolism is often disrupted due to pancreatic cell damage and increased metabolic stress (46). For example, studies have confirmed a close association between plasma tryptophan levels and the occurrence of AP (63). Insufficient supply of essential amino acids can reduce cellular protein synthesis capacity, which in turn negatively affects the repair and regeneration of the pancreas. Meanwhile, the metabolism of non-essential amino acids may become disordered, impacting energy metabolism and antioxidant capacity in acinar cells. A meta-analysis by Xue et al. (64) indicated that glutamine (Gln)-enriched nutritional support is more effective than traditional treatments for SAP. Parenteral Gln supplementation has shown greater effectiveness than enteral supplementation, resulting in increased plasma albumin levels, reduced plasma CRP levels, and lower rates of infection (65).

In summary, the inhibition of protein synthesis, degradation of proteins, and abnormalities in amino acid metabolism are closely linked in the pathogenesis of AP (66). While the regulation of protein synthesis and degradation can help alleviate the ER burden, an imbalance in these processes can exacerbate inflammation and lead to cell death (67). Additionally, abnormal amino acid metabolism exacerbates pancreatic injury through multiple pathways, including impaired cell repair, increased oxidative stress, and immune dysfunction (68). Understanding the interplay between these metabolic processes is crucial for uncovering the pathogenesis of AP and identifying potential therapeutic targets.

## 4 Metabolic interventions and therapeutic approaches

### 4.1 Interventions in lipid metabolism

In recent years, the development of lipase inhibitors and the potential therapeutic effects of lipid metabolism-modulating drugs have garnered significant attention in the context of AP and other metabolic disorders. These interventions, particularly those targeting fatty acid accumulation, oxidative stress, and pancreatic cell protection, have demonstrated considerable promise for clinical application.

#### 4.1.1 Lipase inhibitors

Lipase inhibitors, which modulate lipid metabolism to treat metabolic disorders, have shown potential in the management of various metabolic diseases. In AP, the abnormal activation of lipase and the excessive accumulation of fatty acids are significant factors that exacerbate disease severity. Lipase, a key enzyme catalyzing the hydrolysis of TGs, can intensify lipid peroxidation of pancreatic cell membranes and trigger local inflammatory responses when overactivated. Thus, inhibiting lipase activity has emerged as a potential therapeutic strategy.

Multiple lipase inhibitors have garnered considerable attention in both clinical and experimental studies. Orlistat, a commonly used lipase inhibitor, reduces dietary fat absorption by inhibiting gastrointestinal lipase activity, thus lowering body fat content (69). Studies have shown that Orlistat can alleviate pancreatic injury and inflammatory responses caused by abnormal lipid metabolism to some extent. By reducing excessive fatty acid accumulation, Orlistat can mitigate pancreatic cell damage, decrease fatty acid-induced oxidative stress, and help relieve the condition of AP (70). In a study by Malecki et al. (71), orlistat was administered to obese mice with AP induced by IL-12 and IL-18. The results showed that orlistat significantly decreased intraabdominal fat necrosis compared to the vehicle group (p<0.05). However, it did not affect pancreatic edema, acinar necrosis, or intrapancreatic fat necrosis significantly. This suggests that orlistat may have a certain protective effect on intraabdominal fat necrosis in obese mice with AP, but its effect on pancreatic tissue damage is limited. Clinical data on Orlistat’s efficacy in AP is still limited and requires further validation.

In addition to gastrointestinal lipase inhibitors, those targeting pancreatic lipase have also been considered as potential therapeutic strategies. Pancreatic lipase plays a crucial role in lipid metabolism within the pancreas, and inhibiting its activity can effectively reduce fatty acid production and lipid accumulation in pancreatic cells, thereby decreasing inflammation caused by fatty acids (72). Novel pancreatic lipase inhibitors, such as RABI-767 (73), have demonstrated efficacy in reducing pancreatic injury and inflammatory responses in animal experiments, offering new hope for the treatment of AP. However, their translation to clinical practice remains to be explored.

#### 4.1.2 Fatty acid oxidation modulators

Drugs that improve lipid metabolism, especially those that enhance fatty acid oxidation, have become a focal point in research. Fatty acids are key contributors to pancreatic injury and inflammatory responses. Medications that regulate fatty acid oxidation can effectively minimize their accumulation in pancreatic tissue, thereby alleviating the oxidative stress and inflammation associated with fatty acids. Conjugated linoleic acid, a common regulator of fatty acid oxidation, promotes fatty acid oxidation and reduces fat accumulation. In animal models of AP, conjugated linoleic acid has been shown to reduce excessive fatty acid accumulation, lessen pancreatic inflammation, and improve pancreatic cell function (74).

Moreover, studies have shown that modulating the AMP-activated protein kinase (AMPK) pathway can effectively promote fatty acid oxidation, decrease fatty acid accumulation, reduce pancreatic inflammation, and improve the condition of AP (75). AMPK agonists, such as AICAR (76), have been proven to alleviate fatty acid metabolic disorders caused by AP in animal models and hold potential for clinical application. Another approach is to regulate fatty acid metabolism by activating peroxisome proliferator-activated receptors (PPARs). PPAR-α, an important regulator of fatty acid metabolism in the pancreas, has been found to promote fatty acid oxidation and reduce fatty acid accumulation when its receptor is activated (77). PPAR-α agonists, such as Fenofibrate, have shown in animal experiments to reduce fatty acid deposition in pancreatic tissue and significantly alleviate inflammatory responses (78). Additionally, PPAR-γ receptor agonists have demonstrated in some studies the ability to improve fat metabolism and mitigate AP (79). However, clinical trials evaluating these agents in AP are still in early stages, and their efficacy and safety in humans need to be further established.

These findings have shown that interventions targeting lipid metabolism, including lipase inhibitors and fatty acid oxidation modulators, have shown promise in mitigating the severity of AP. Future research should continue to explore the mechanisms of these interventions and their potential clinical applications to develop more effective therapeutic strategies for AP.

### 4.2 Interventions in glucose metabolism

In the management of AP, insulin therapy and glycemic control are essential therapeutic strategies, as hyperglycemia and IR are critical factors that exacerbate pancreatic injury and promote inflammatory responses. AP is frequently associated with IR and hyperglycemia, particularly in severe cases, where pancreatic function is compromised, leading to reduced insulin secretion capacity. Additionally, the systemic inflammatory response can induce stress hyperglycemia, further worsening pancreatic damage. Therefore, rational control of hyperglycemia and improvement of IR hold significant clinical importance in the treatment of AP.

#### 4.2.1 Insulin therapy and glycemic control

The occurrence of hyperglycemia in AP is not only directly related to pancreatic dysfunction but also closely associated with systemic stress responses. Due to impaired insulin secretion by the pancreas, patients with pancreatitis often experience a degree of insulin deficiency, which exacerbates hyperglycemia. Insulin therapy plays a crucial role in managing hyperglycemia in AP. Studies have shown that insulin can effectively regulate blood glucose levels, reduce inflammatory responses, and improve pancreatic function. In the early stages of AP, maintaining blood glucose within normal or near-normal ranges through rational insulin therapy helps prevent further damage to the pancreas and other organs caused by glucose fluctuations (80). Insulin’s effects extend beyond glycemic control; it can also inhibit hepatic gluconeogenesis, promote glucose utilization by peripheral tissues, and enhance cellular insulin sensitivity, thereby further reducing IR and improving the patient’s metabolic state (81). In clinical practice, insulin therapy typically includes intravenous insulin infusion and subcutaneous insulin injections. Intravenous insulin therapy is commonly used in severe cases of AP, as these patients often have significant hyperglycemia and require rapid and precise glycemic control (82). Intravenous insulin can quickly take effect, stabilize blood glucose levels, and avoid excessive or insufficient glucose fluctuations. For mild or stable cases of AP, subcutaneous insulin injections are usually effective in controlling blood glucose and are more convenient to administer. The dose and regimen of insulin should be individually adjusted based on the patient’s blood glucose levels and the severity of pancreatitis. Glycemic control not only improves IR but also reduces adverse effects caused by hyperglycemia, such as metabolic disorders, immune suppression, and tissue oxidative damage. Studies have shown that controlling blood glucose can reduce pancreatic injury, shorten hospital stays, lower the incidence of complications, and significantly improve the prognosis of patients with AP (83). Therefore, early detection and timely correction of hyperglycemia are crucial in the treatment of AP.

Insulin lowers serum TG levels by stimulating LPL activity. LPL, produced by capillary endothelial cells in muscles and adipose tissues, hydrolyzes TG into glycerol and fatty acids (84). Insulin triggers LPL activity, metabolizing TG-rich lipoproteins such as chylomicrons and very low-density lipoprotein (VLDL), thus reducing serum TG levels. In a retrospective study on 12 hypertriglyceridemic pancreatitis (HTG-AP) patients, serum TG levels dropped to below 500 mg/dL within 2–3 days of insulin therapy, with no complications and good clinical outcomes (85). A comparative review of 34 HTG-AP cases showed that insulin therapy effectively reduced TG levels, with most patients recovering after 3–5 days of treatment (86).

In addition to lowering TG levels, insulin can inhibit inflammatory responses in HTG-AP. When TG levels are high, pancreatic enzymes break down TG into toxic FFA, inducing inflammation. Insulin reduces TG levels, decreases FFA production, and suppresses inflammatory mediator release, thereby alleviating inflammation (87). A study found that insulin therapy lowers serum CRP, an inflammation marker, indicating its anti-inflammatory effects. Insulin therapy may also improve pancreatic function (88). In HTG-AP, pancreatic inflammation and injury can lead to functional impairment. By reducing inflammation and lowering TG levels, insulin protects pancreatic tissue and promotes functional recovery (88). For example, after insulin therapy, serum amylase and lipase levels gradually normalize, indicating improved pancreatic secretory function (89). Insulin may also promote pancreatic cell repair and regeneration.

Insulin therapy is suitable for all HTG-AP patients, with diabetic patients benefiting from simultaneous glucose and TG control. Insulin is administered intravenously at 0.1-0.3 units/kg/h, with blood glucose monitoring to avoid hypoglycemia. When blood glucose falls below 200 mg/dL, 5% dextrose solution is co-infused. TG levels are regularly monitored to assess therapeutic effects (90). Numerous studies support insulin therapy for HTG-AP. Future research could explore optimal insulin doses, timing, and treatment durations. More randomized controlled trials (RCT) are needed to clarify the role and mechanism of insulin therapy in HTG-AP, providing stronger evidence for clinical practice.

#### 4.2.2 Interventions for IR

IR is a common metabolic abnormality in AP, particularly in severe cases. It plays a key role in leading to hyperglycemia and metabolic disorders. The development of IR occurs due to impaired insulin signaling pathways, which hinder insulin’s ability to effectively promote glucose uptake and metabolism, resulting in elevated blood glucose levels.

Currently, intervention strategies for IR can be categorized into pharmacological and non-pharmacological approaches. In terms of pharmacological interventions, several medications have been widely used and have demonstrated significant efficacy. Metformin, a well-established treatment for Type 2 diabetes, has increasingly been applied to address IR in recent years (91). In clinical practice, metformin has been used to treat many patients with mild to moderate AP complicated by obesity and IR. Clinical research indicates that metformin effectively improves IR by inhibiting hepatic gluconeogenesis, enhancing peripheral tissue insulin sensitivity, and reducing fatty acid release from adipocytes (92). One clinical trial involving a large cohort of patients with mild to moderate AP, obesity, and IR found that metformin treatment led to significant decreases in fasting blood glucose levels, 2-hour postprandial glucose levels, and HOMA-IR scores, alongside improvements in lipid profiles (6). The rate of adverse reactions was acceptable, primarily consisting of mild gastrointestinal issues that most patients could tolerate.

Thiazolidinediones, such as rosiglitazone and pioglitazone, have also been used to treat AP patients with metabolic syndrome and IR. These drugs work by activating PPAR-γ, which regulates fat tissue metabolism and improves adipocyte function to enhance insulin action (93). Clinical studies have shown that after several weeks of treatment with thiazolidinediones, patients exhibited significant improvements in insulin sensitivity markers, as well as correction of lipid metabolic abnormalities, such as reduced TG levels and increased high-density lipoprotein (HDL) cholesterol (44). Furthermore, observations related to AP disease indicators revealed that patients receiving these medications experienced a quicker reduction in pancreatic inflammatory markers like amylase and lipase and exhibited less severe pancreatic tissue pathology, demonstrating potential protective effects on the pancreas (94).

Glucagon-like peptide-1 (GLP-1) receptor agonists, including liraglutide and saxagliptin, are emerging antidiabetic drugs that show promise in improving IR in AP patients. Clinical trials targeting AP patients with cardiovascular risk factors have indicated that GLP-1 receptor agonists lead to more precise blood glucose control, reduced fluctuations in blood glucose levels, and a steady decrease in glycated hemoglobin levels (95). Ongoing research has suggested that these drugs not only enhance insulin sensitivity but also exhibit anti-inflammatory properties and promote pancreatic cell repair (96). Researchers have observed significant reductions in inflammatory markers such as CRP and IL-6 in patients receiving GLP-1 receptor agonists, indicating their ability to mitigate inflammatory responses in AP (97). Additionally, these drugs have demonstrated potential in activating pancreatic cell repair mechanisms.

In conclusion, these drugs have shown promise for treating AP patients with IR. Ongoing research is expected to further refine treatment strategies and improve patient outcomes in the future.

#### 4.2.3 Combined insulin and heparin therapy in hyperlipidemic pancreatitis

For patients with hyperlipidemic pancreatitis, combined insulin and heparin therapy is a clinically used approach for rapid TG reduction (98). This therapy is particularly effective in patients with severe hypertriglyceridemia (serum TG levels >1,000 mg/dl), where the goal is to lower TG levels and reduce pancreatic injury (99). Insulin activates LPL, an enzyme that accelerates the degradation of chylomicrons into glycerol and FFA, thereby lowering serum TG levels (100). Heparin stimulates the release of endothelial LPL into circulation, further enhancing the breakdown of TG-rich lipoproteins. Together, insulin and heparin work synergistically to promote TG metabolism and alleviate the metabolic burden on the pancreas (101).

Studies demonstrated that combined insulin and heparin therapy can significantly reduce serum TG levels within a short period. For example, Berger et al (102). used continuous infusions of insulin and heparin in patients with hyperlipidemic pancreatitis and observed rapid declines in TG levels. Some case reports have documented the use of intravenous heparin (5000U twice daily) combined with intravenous insulin and achieved similar results (103). The dosage and route of administration may vary, but the goal is to maintain TG levels below 500 mg/dl to expedite clinical improvement (104).

While combined therapy is effective, there are some controversies regarding heparin use. Heparin can initially increase circulating LPL levels, but prolonged use may lead to depletion of LPL stores in the liver, potentially causing a rebound effect (105). Animal models have confirmed that heparin accelerates the transport of LPL to the liver, long-term use can result in LPL deficiency and may need to be carefully considered (106). Despite these concerns, the short-term use of heparin in combination with insulin remains a valuable option for rapidly reducing TG levels in acute settings.

In summary, the regulation of glucose metabolism is of vital importance in the treatment of AP. Insulin therapy and glycemic control are key strategies to prevent further pancreatic and organ damage, improve IR, and enhance patient outcomes. Intervention strategies for IR, including pharmacological and non-pharmacological approaches, have shown potential in improving IR and reducing metabolic disorders. Combined insulin and heparin therapy has demonstrated efficacy in rapidly lowering TG levels in hyperlipidemic pancreatitis. However, research into these therapeutic interventions is still ongoing, and optimizing dosing regimens and understanding long-term effects remain crucial areas for future exploration.

### 4.3 Antioxidant therapy

In the management of AP, the control of oxidative stress is an essential component, as it not only exacerbates pancreatic injury but also promotes the development and progression of metabolic disturbances, thereby further intensifying the inflammatory response. Antioxidants, such as vitamin C and vitamin E, have garnered increasing attention in the treatment of AP due to their ability to mitigate oxidative damage. By reducing the generation of free radicals and enhancing antioxidant defense mechanisms, these agents play a significant role in alleviating oxidative stress, improving metabolic disturbances, and inhibiting inflammatory responses.

#### 4.3.1 Oxidative stress responses in AP

In the pathogenesis of AP, the generation of ROS, and the disruption of redox homeostasis are pivotal factors. AP is often triggered by acute inflammatory responses within pancreatic tissue, where ROS serve as critical modulators of cellular stress, contributing to various aspects of pancreatic injury (107). Under normal physiological conditions, ROS are natural byproducts of cellular metabolism, primarily generated through the mitochondrial electron transport chain. However, during AP, ROS production is significantly increased due to cellular injury or stress (108). Elevated ROS levels can induce lipid peroxidation, protein denaturation, and direct DNA damage, ultimately triggering apoptosis or necrosis (109). In AP, both inflammatory cells (such as neutrophils and macrophages) and damaged pancreatic cells themselves produce substantial amounts of ROS (110). These ROS activate a cascade of signaling pathways, further exacerbating the inflammatory response and leading to pancreatic tissue damage (7). The role of oxidative stress in AP is twofold. First, oxidative stress promotes the initiation and amplification of inflammatory responses by activating various signaling pathways (111).

ROS can activate transcription factors such as nuclear factor-κB (NF-κB) and activator protein-1 (AP-1) (112), thereby inducing the expression of pro-inflammatory cytokines, including tumor necrosis factors and interleukins. These cytokines attract additional immune cells to the site of inflammation, intensifying pancreatic injury. Second, oxidative stress induces necrosis or apoptosis by damaging lipids, proteins, and DNA within pancreatic cells (113). Excessive ROS targets the cell membranes of pancreatic cells, initiating lipid peroxidation reactions that compromise membrane integrity and lead to cell death. ROS can oxidize proteins and DNA, disrupt their normal biological functions, and induce genetic mutations or damage, further exacerbating pancreatic injury (114). Moreover, redox imbalance impacts the autophagy process in the pancreas (115). Autophagy is a critical metabolic pathway that degrades damaged organelles and proteins to maintain cellular homeostasis. In AP, excessive ROS not only inhibits the normal function of autophagy but may also alter autophagy regulation by activating autophagy-related genes, such as glutamic oxaloacetic transaminase, leading to the accumulation of damaged materials within cells and exacerbating pancreatic injury (116). In summary, there is a close relationship between redox imbalance and the progression of AP. Excessive ROS generation and oxidative stress exacerbate pancreatic cell injury, promote inflammatory responses, and ultimately lead to extensive pancreatic tissue damage and functional failure (117). Therefore, modulating ROS generation and restoring redox balance may represent a promising therapeutic strategy for AP. The use of antioxidants or modulation of antioxidant enzyme activity may help mitigate oxidative stress-induced pancreatic damage and improve clinical outcomes.

Controlling oxidative stress is an essential component in managing AP, as it not only exacerbates pancreatic injury but also promotes the development and progression of metabolic disturbances, thereby intensifying the inflammatory response (118). Antioxidants, such as vitamin C and vitamin E, have garnered increasing attention in the treatment of AP due to their ability to mitigate oxidative damage (119). By reducing the generation of free radicals and enhancing antioxidant defense mechanisms, these agents play a significant role in alleviating oxidative stress, improving metabolic disturbances, and inhibiting inflammatory responses.

#### 4.3.2 Vitamin C

Vitamin C and vitamin E are two widely recognized antioxidants used in clinical practice. Vitamin C, being a water-soluble antioxidant, can directly scavenge free radicals, which helps reduce intracellular oxidative damage, particularly lipid peroxidation (120). In AP, pancreatic tissue often undergoes severe oxidative stress due to excessive free radicals, such as superoxide anions, hydrogen peroxides, and hydroxyl radicals. This oxidative stress leads to lipid peroxidation of cell membranes, DNA damage, and protein denaturation, all of which worsen pancreatic injury and inflammatory responses. Vitamin C protects pancreatic cells from oxidative damage by inhibiting the generation of harmful free radicals, thereby reducing inflammation and improving the conditions. Clinical and animal studies have shown that vitamin C supplementation can significantly improve the prognosis of patients with AP, especially in severe cases, by reducing oxidative stress and organ damage (121). Vitamin C also regulates immune responses, reduces cellular inflammation and immune activation, thereby lowering the risk of multi-organ failure (122). Additionally, vitamin C enhances the antioxidant capacity of endothelial cells, improving microcirculatory blood flow and effectively alleviating pancreatic ischemic injury caused by AP (123).

In recent years, numerous clinical studies have explored the efficacy of vitamin C in the treatment of AP. For instance, a prospective cohort study involving 43 patients with mild to moderate AP found that those who received vitamin C supplementation (1–2 g/day) experienced a significant reduction in serum amylase and lipase levels within three days of treatment. The average hospital stay for these patients was shortened by approximately two days compared to the control group (123). Another RCT with 84 SAP patients revealed that high-dose vitamin C infusion (3–6 g/day) administered over 7–14 days was linked to a 30% lower incidence of pancreatic necrosis and a 20% reduction in the risk of systemic inflammatory response syndrome (SIRS) (124). Furthermore, in a multicenter study involving 418 patients with AP, vitamin C supplementation not only improved pancreatic outcomes but also enhanced overall recovery (121). Patients receiving vitamin C had a 25% lower rate of infectious complications and a 15% decrease in the need for intensive care unit (ICU) admission. Collectively, these studies highlight the therapeutic potential of vitamin C in managing AP and its complications. However, it is worth noting that the optimal dose and duration of vitamin C administration may vary depending on the severity of the disease and individual patient factors. Further large-scale, well-designed clinical trials are needed to establish standardized treatment protocols for vitamin C in AP.

#### 4.3.3 Vitamin E

Vitamin E, a lipid-soluble antioxidant, primarily protects cell membranes from oxidative damage by inhibiting lipid peroxidation. Patients with AP often exhibit increased lipid peroxidation, and vitamin E can help reduce cell membrane damage by decreasing the formation of peroxidized lipids, thereby minimizing pancreatic cell injury. Clinically, vitamin E has been shown to lower the release of inflammatory mediators and the infiltration of inflammatory cells, thereby alleviating pancreatic tissue inflammation (125). Moreover, vitamin E enhances antioxidant capacity by regulating the intracellular antioxidant enzyme system (such as glutathione peroxidase and superoxide dismutase), further reducing oxidative stress-induced pancreatic injury (126).

Multiple clinical studies have demonstrated the efficacy of vitamin E in treating AP. For example, a multicenter prospective cohort study involving 39 patients with AP found that those receiving vitamin E supplementation (400–800 IU/day) showed a significant reduction in serum levels of inflammatory markers such as CRP and IL-6 within five days of treatment. The average severity score of pancreatitis in these patients dropped by approximately 1.2 points compared to the control group (127). An RCT with 100 SAP patients revealed that combination therapy with vitamin E (400 IU/day) and vitamin C (2 g/day) over 10–14 days was associated with a 35% lower incidence of pancreatic necrosis and a 25% reduction in the risk of SIRS (128). Additionally, histopathological examination of pancreatic tissue from animal models of AP confirmed that vitamin E could reduce pancreatic tissue edema, inflammatory cell infiltration, and acinar cell necrosis (126). Collectively, these studies highlight the therapeutic potential of vitamin E in managing AP and its complications. However, it is worth noting that the optimal dose and duration of vitamin E administration may vary depending on the severity of the disease and individual patient factors. Further large-scale, well-designed clinical trials are needed to establish standardized treatment protocols for vitamin E in AP.

In summary, the use of antioxidants such as vitamin C and vitamin E in AP is of significant clinical importance. These agents reduce oxidative stress, improve metabolic disturbances, mitigate lipid and glucose metabolic disorders, help alleviate pancreatic injury, inhibit inflammatory responses, and potentially prevent complications and disease progression in AP. Antioxidant supplementation in the treatment of AP has therapeutic value, but further clinical research is needed to verify its long-term effects and optimal usage protocols. Future studies should focus on elucidating the mechanisms of action of these antioxidants and exploring their potential synergistic effects with other therapeutic interventions to optimize clinical outcomes in patients with AP.

### 4.4 Emerging therapies

Recent advances have provided insights into novel therapeutic approaches for acute AP, particularly in the areas of metabolic regulation and gut microbiome modulation. Fibroblast growth factor 21 (FGF21) analogs have emerged as promising candidates for treating AP. FGF21 is a metabolic hormone that affects glucose and lipid metabolism, as well as insulin sensitivity. Preclinical studies have shown that FGF21 alleviates cerulein-induced AP by activating the Sirt1-autophagy signaling pathway (129). This activation aids in repairing damaged mitochondria and lysosomes, reducing autophagic damage, and mitigating systemic inflammation (130). Furthermore, FGF21 demonstrates protective effects against pancreatic fibrosis and inflammation. Moreover, mRNA-based therapies that deliver FGF21 and apolipoprotein A1 (APOA1) have been explored as innovative treatments for AP. These therapies utilize liver-targeted lipid nanoparticles (LNPs) to facilitate the production of endogenous proteins, resulting in sustained therapeutic effects (131). Experimental studies indicate that these therapies can reduce pancreatic injury and improve histological outcomes in models of AP.

In parallel, gut microbiome modulation has gained significant attention due to its critical role in the pathogenesis of AP. The condition is often associated with intestinal dysbiosis, characterized by a decrease in beneficial bacteria such as Bifidobacteria, and an increase in potentially harmful bacteria like Enterococcus (132). Strategies such as probiotics or fecal microbiota transplantation have shown promise in restoring intestinal barrier function and reducing systemic inflammation in patients with AP (133). Probiotics can enhance the epithelial barrier and modulate the composition of commensal microbiota, while fecal microbiota transplantation can help reconstruct normal intestinal flora, aiding in the treatment of both intestinal and extraintestinal diseases (134).

In summary, these emerging therapies reflect the multifaceted nature of AP treatment and offer new possibilities for improving patient outcomes. FGF21 analogs and gut microbiome modulation are pivotal in advancing the management of this complex disease.

### 4.5 Challenges of metabolic disturbances in the treatment of AP

The metabolic profiles of patients with AP are highly heterogeneous, demonstrating significant individual variability. This heterogeneity poses a substantial challenge for developing standardized therapeutic protocols for metabolic interventions (**Table 1**). Given this complexity, a precision medicine approach is essential. This approach tailors treatment strategies to the specific metabolic status of each patient. For instance, patients experiencing severe lipid metabolic disturbances may benefit from fatty acid metabolism modulators (135), while those with abnormal glucose metabolism might require insulin therapy or insulin sensitizers (136).

**Table 1:** Current metabolic regulation therapies for acute pancreatitis.

Intervention strategies	Targeted therapies	Models	Mechanisms	References
Lipase Inhibitors	Orlistat	obese AP mice	Inhibiting gastrointestinal lipase activity and decreasing excessive fatty acid accumulation.	(71)
	RABI-767	SAP patients	Reducing fatty acid production and lipid deposition in pancreatic cells, thereby decreasing inflammation caused by fatty acids.	(73)
Fatty Acid Oxidation Modulators	Conjugated linoleic acid	SAP mices	Reducing excessive fatty acid accumulation and mitigating pancreatic inflammation.	(74)
	AMPK agonists	obese AP mice	Activating of AMPK signalling pathway, alleviating pancreatic acinar cell necroptosis and converting SAP to MAP in obese mice.	(76)
	PPARs agonists	SAP mices	Improving fat metabolism and mitigating AP.	(78, 79)
Insulin therapy and glycemic control	Insulin	HTG-AP patients	Lowering TG levels, inhibiting inflammatory responses in HTG-AP.	(85, 86, 88–90)
Interventions for IR	Metformin	patients with mild to moderate AP complicated by obesity and IR	Enhancing peripheral tissue insulin sensitivity and reducing fatty acid release from adipocytes.	(6)
	Thiazolidinediones	AP patients with metabolic syndrome and IR	Activating PPAR-γ, thereby regulating fat tissue metabolism and improving adipocyte function.	(44, 94)
	GLP-1 receptor agonists	AP patients with cardiovascular risk factors	Enhancing insulin sensitivity but also exhibiting anti-inflammatory properties and promoting pancreatic cell repair.	(97)
Combined insulin and heparin therapy	Combined insulin and heparin therapy	patients with hyperlipidemic pancreatitis	Insulin and heparin working synergistically to promote the metabolism of TGs and alleviating the metabolic burden on the pancreas.	(98, 99, 102–104)
Antioxidant therapy	Vitamin C	AP patients	Inhibiting the generation of these harmful free radicals, thereby reducing pancreatic inflammation and improving the condition.	(121–124)
	Vitamin E	AP patients and AP mices	Enhancing antioxidant capacity by regulating the intracellular antioxidant enzyme system.	(125–128)
Emerging therapies	FGF21 analogs	caerulein-induced AP mices	Protecting pancreatic from fibrosis and inflammation.	(131)
	Gut microbiome modulation	AP patients	Restoring intestinal barrier function and reducing systemic inflammation in AP patients.	(133, 134)

In the realm of precision medicine for AP, biomarkers play a pivotal role. Fatty acid profiles can serve as key biomarkers. By analyzing the composition and ratios of different fatty acids in patients, clinicians can gain insights into the lipid metabolic state (137). For example, elevated levels of certain saturated fatty acids could indicate a heightened risk of lipotoxicity, which would guide the appropriate use of fatty acid metabolism modulators (138). Oxidative stress markers are another crucial category of biomarkers, reflecting the balance between oxidative and anti-oxidative mechanisms in the body. Increased levels of oxidative stress markers may suggest ongoing tissue damage and inflammation, assisting in determining the timing and intensity of antioxidant therapy as part of metabolic interventions (139). Future research should focus on designing personalized treatment plans based on these biomarkers to enhance therapeutic efficacy, minimize adverse effects, and maximize the success rate of AP treatments.

Despite the promising potential of metabolic regulation in treating AP, many questions remain unanswered. Future studies should further explore the intricate relationship between metabolic disturbances and AP, emphasizing the underlying mechanisms of various metabolic pathways, including lipid, glucose, and amino acid metabolism. Additionally, researchers should investigate the correlation between metabolic regulation and clinical manifestations of AP, examining how different metabolic disturbances relate to disease progression, complications, and prognosis.

Finally, addressing the challenges of translating findings from animal models to human trials is important. Species-specific metabolic differences can significantly impact the outcomes of metabolic interventions. For example, rodents, which are commonly used in animal studies, possess distinct lipid metabolism patterns compared to humans, potentially leading to discrepancies in the efficacy and safety profiles of metabolic modulators as research transitions from animal models to human applications (140). Understanding and accounting for these species-specific metabolic differences is crucial for the successful translation of pre-clinical findings into effective clinical therapies for patients with AP.

Through systematic and comprehensive research into metabolic mechanisms, a more precise theoretical basis can be established to guide clinical practice and improve patient outcomes in the treatment of AP.

## 5 Conclusion

The accumulation of fatty acids, dysfunction in amino acid metabolism, and disorders in glucose metabolism are the common metabolic disturbances associated with AP. These factors can exacerbate pancreatic injury and promote inflammation. Specifically, the accumulation of fatty acids not only directly activates inflammatory responses but also disrupts cell membranes and increases oxidative stress, which further worsens pancreatic injury. Furthermore, hyperglycemia and IR intensify pancreatic damage and inflammation by enhancing oxidative stress and fatty acid metabolic disturbances.

The interventions for metabolic regulation in the treatment of AP have been extensively explored. Interventions aimed at lipid metabolic disturbances, such as lipase inhibitors and fatty acid oxidation modulators, have shown promise in mitigating pancreatic injury. Insulin therapy has proven effective in managing hyperglycemia, potentially slowing the inflammatory process by reducing the burden on the pancreas. The use of antioxidants, such as vitamin C and vitamin E, may also help alleviate oxidative stress and protect pancreatic cells. However, despite the significant potential of metabolic regulation in treating AP, the underlying mechanisms need further clarification.

Future research should focus on the complex relationship between metabolic disturbances and AP, particularly the correlation between metabolic profiles, disease progression, and clinical outcomes. Personalized treatments and the development of drugs targeting specific metabolic pathways should also be prioritized as key directions for future therapies. Through these studies, more precise and effective intervention strategies may be developed for the clinical management of AP.

In conclusion, metabolic disturbances play a crucial role in the pathogenesis, progression, and clinical manifestations of AP. Metabolic regulation not only represents a promising therapeutic approach but also offers a broad framework for future research initiatives.
